# Key Strategies for Clinical Management and Improvement of Healthcare Services for Cardiovascular Disease and Diabetes Patients in the Coronavirus (COVID-19) Settings: Recommendations From the REPROGRAM Consortium

**DOI:** 10.3389/fcvm.2020.00112

**Published:** 2020-06-16

**Authors:** Sonu Bhaskar, Aarushi Rastogi, Vijay Kumar Chattu, Anil Adisesh, Pravin Thomas, Negman Alvarado, Anis D. Riahi, Chakrakodi N. Varun, Anupama R. Pai, Sarah Barsam, Antony H. Walker

**Affiliations:** ^1^Pandemic Health System REsilience PROGRAM (REPROGRAM) Consortium, CVD and Metabolic REPROGRAM Sub-committee^†^, Sydney, NSW, Australia; ^2^Liverpool Hospital & South West Sydney Local Health District (SWSLHD), Department of Neurology & Neurophysiology, Sydney, NSW, Australia; ^3^Neurovascular Imaging Laboratory, Ingham Institute for Applied Medical Research, Clinical Sciences Stream, Sydney, NSW, Australia; ^4^NSW Brain Clot Bank, NSW Health Statewide Biobank, Sydney, NSW, Australia; ^5^South Western Sydney Clinical School, University of New South Wales (UNSW), Sydney, NSW, Australia; ^6^Department of Medicine, St. Michael's Hospital, University of Toronto, Toronto, ON, Canada; ^7^University Hospitals Birmingham NHS Foundation Trust, Birmingham, United Kingdom; ^8^Department of Neurophysiology, Instituto Médico Dr. Rodriguez Alfici, Godoy Cruz, Argentina; ^9^Department of Neurology, Faculty of Medicine, Military Hospital of Tunis, University of Tunis El Manar, Tunis, Tunisia; ^10^State Level Virus Research and Diagnostics Laboratory, Bangalore Medical College and Research Institute, Bengaluru, India; ^11^Department of Neuromicrobiology, National Institute of Mental Health and Neurosciences (NIMHANS), Bengaluru, India; ^12^Department of Hematology, North Middlesex Hospital, King's Thrombosis Center & King's College Hospital NHS Foundation Trust, King's College London, London, United Kingdom; ^13^Department of Cardiothoracic Surgery, Lancashire Cardiac Centre, Blackpool Victoria Hospital, NHS, Blackpool, United Kingdom

**Keywords:** coronavirus disease 2019 (COVID-19), healthcare services, cardiovascular diseases (CVDs), diabetes, obesity, personal protective equipment (PPE), clinical algorithm

## Abstract

Patients with cardiovascular disease and diabetes are at potentially higher risk of infection and fatality due to COVID-19. Given the social and economic costs associated with disability due to these conditions, it is imperative that specific considerations for clinical management of these patients be observed. Moreover, the reorganization of health services around the pandemic response further exacerbates the growing crisis around limited access, treatment compliance, acute medical needs, and mental health of patients in this specific subgroup. Existing recommendations and guidelines emanating from respective bodies have addressed some of the pressure points; however, there are variations and limitations *vis a vis* patient with multiple comorbidities such as obesity. This article will pull together a comprehensive assessment of the association of cardiovascular disease, diabetes, obesity and COVID-19, its impact on the health systems and how best health systems can respond to mitigate current challenges and future needs. We anticipate that in the context of this pandemic, the cardiovascular disease and diabetes patients need a targeted strategy to ensure the harm to this group does not translate to huge costs to society and to the economy. Finally, we propose a triage and management protocol for patients with cardiovascular disease and diabetes in the COVID-19 settings to minimize harm to patients, health systems and healthcare workers alike.

## Introduction

On March 11th, 2020, coronavirus disease 2019 (COVID-19) was designated as a global pandemic by the World Health Organization (WHO). As of 28th May, 5,716,271 confirmed cases have been reported with ~356,124 deaths globally from 188 countries (1). In a matter of months, this has escalated into an unprecedented public health as well as an economic crisis. Several studies have confirmed that patients with COVID-19 show distinctive and relatively significant comorbidities of diabetes, obesity and cardiovascular disease (CVD) (2–12). Furthermore, COVID-19 patients with diabetes, obesity and CVD conditions are at a higher risk of morbidity and mortality (6, 7, 13). Conversely, patients with diabetes, CVD and obesity are also at a higher risk of contracting COVID-19 infection (6, 7, 14). Broadly speaking, CVD, diabetes and obesity are associated with poor clinical outcomes (15, 16). Therefore, in the milieu of COVID-19; public health systems, carers, and healthcare providers must take appropriate measures to mitigate the infection risks in this population and consider health system reorganization and adoption of technologies to sustain ongoing management (17–19). The frontline healthcare workers triaging and managing COVID-19 patients should consider various risks and their compounding effects on the prognoses of patients with CVD, diabetes and/or obesity.

## Risks and Outcomes of COVID-19 in This Population

Independent of other medical problems such as CVD, patients with diabetes are at elevated risk for infection from COVID-19 by 2-to-3 fold (13). This may be attributed to the reduced functioning of the immune system caused by high blood glucose levels (13). Moreover, diabetes is often accompanied by CVD, obesity and old age, all of which are known to increase the risk of infection (14). Outcomes of infection by COVID-19 are also poor in this population. Of 72,314 patients from the Chinese Center for Disease Control and Prevention case series, case fatality rate (CFR) was elevated among those with diabetes and CVD; 7.3 and 10.5% respectively compared to an overall CFR of 2.3% (5). Outcomes are worse in people with poorly controlled diabetes, and in those with additional chronic medical conditions such as CVD and obesity (7, 13).

A recent meta-analysis of eight studies from China including 46,248 infected patients showed the most prevalent comorbidities were high blood pressure (17 ± 7%, 95% CI 14–22%) and diabetes (8 ± 6%, 95% CI 6–11%), followed by CVD (5 ± 4%, 95% CI 4–7%) (6). At this time, though the mechanism of these associations remains unclear, the potential explanations include CVD being more prevalent in those with advancing age, a functionally impaired immune system, elevated levels of angiotensin-converting enzyme 2 (ACE2), or a predisposition to COVID-19 for those with CVD (6). There is significant overlap in risk factors of CVD and venous thromboembolism (VTE); with CVD risk factors such as older age, smoking, and adiposity associated with high VTE risk (20). A recent Chinese study on 1026 COVID-19 patients reported that 40% (*n* = 407) at high risk of venous thromboembolism; and the high-risk patients who didn't receive prophylactic therapy (11%) developed venous thromboembolism (21).

As of 4th April 2020, the Intensive Care National Audit and Research Centre (ICNARC) in the United Kingdom (UK) received notification of 2,621 COVID-19 positive cases requiring critical care (22). Analysis of this data suggest a significantly greater number of COVID-19 positive cases, than “seasonal” non-COVID viral pneumonia patients, were obese, with body mass index (BMI) ≥30 kg/m^2^ (38% compared to 31%, chi-square 28.2, *p* < 0.00001). The requirement for ventilatory support was equal between the obese and non-obese patients (76 and 68% of cases respectively, *p* = 0.077). Obesity was associated with higher mortality rates in critical care when compared to normal or underweight COVID-19 positive patients (58% compared to 45%, chi-square 8.3 *p* = 0.004). These data derive from the ICNARC case mix programme database. The case mix programme is the national clinical audit of patient outcomes from adult critical care coordinated by the ICNARC. For more information on the representativeness and quality of these data, we encourage readers to contact ICNARC (22).

## Gaps, Challenges, and Concerns About the Management of CVD and Diabetes

In the setting of COVID-19, specialist cardiologists and endocrinologists are confronted with a number of critical issues on management and treatment of CVD. There has been speculation regarding the risk associated with the use of ACE inhibitors (ACEi) and angiotensin receptor blockers (ARBs) in patients with COVID-19 (23, 24). This is particularly relevant to patients with diabetes and CVD, many of whom rely on such pharmacotherapy for the treatment of retinopathy, nephropathy and hypertension (14). Though the ACEi and ARB are commonly used in the management of CVDs (hypertension, coronary artery disease, congestive heart failure) and diabetes, there are conflicting data from studies (13, 15, 16) demonstrating an increase or having minimal effect on ACE2 levels (25–29).

Poor glycaemic index is known to cause immune suppression through impaired neutrophil degranulation, deficient complement system and phagocytosis (30). The co-existence of CVD and diabetes is a known risk factor for several serious respiratory viral illnesses such as Influenza (31). With poor glycaemic control being correlated with worse prognosis in diabetic patients infected with COVID-19, glucose control is key to the prevention of infection and minimizing the severity of and morbidity caused by infection (32). However, the swift transition of primary health care provision from in-person to teleconsultations has led to many patients being unable to access services for regular check-ups, presumably due to lack of literacy and access to appropriate technology. Moreover, an increasing number of physicians have reported a sudden decrease in the incidence of myocardial infarction, stroke, and other acute conditions (33, 34). Given that the prevalence of these conditions would be invariably the same, if not elevated, in these circumstances, this indicates a problematic decline in the number of patients presenting to hospital with these critical conditions. Likewise, there are increasing concerns related to the postponement of elective cardiac and vascular surgeries. With pressure building on the available beds, it is imminent that only a select group of patients with clinical indication in which surgery cannot be postponed will receive the therapy (35). Furthermore, impact of postponement on those who will eventually receive prolonged and delayed surgery vis a vis their long-term morbidity is not known. In patients with diabetes, CVD or obesity, physical exercise is critical to improving patient outcomes (36). With the implementation of self-isolation however, the ability and motivation to engage in physical exercise are greatly diminished.

Healthcare workers responsible for the care of patients with diabetes and cardiovascular disorders infected with COVID-19 face threats to their own well-being, being at risk of exposure to a high viral load (37). Time is critical in acute myocardial or cerebral infarction. Given the reorganization of healthcare services, additional pressure on frontline services for COVID-19 cases, repurposing of other physicians to meet the demand, additional resources limitations are being realized across the spectrum in delivering time-critical reperfusion therapy (34). It is more likely that reperfusion services will also have time-constrained service hours, and due to palpable risks from COVID-19 positive patients to healthcare workers delivering reperfusion therapy, there will be significant negative impact and delays in reperfusion therapy. All patients with acute neuro/cardiovascular events, including acute myocardial infarction (AMI) and acute ischemic stroke (AIS) may be recommended to follow the overarching COVID-19 protocol to screen for any positive cases in order to minimize the risk to healthcare workers (34).

Refugees, undocumented immigrants and members of aboriginal communities also have limited provisions of access and medical relief in pandemic situations, due to structural factors and poor socioeconomic conditions that put them at compounded risk due to cardiovascular and diabetes comorbidities.

## Existing Recommendations and Guidelines for Diabetic Patients

Professional societies such as the American Association of Clinical Endocrinologists (38) and European society of endocrinology (39) are in agreement on the need for people with diabetes to prevent and prepare for the spread of COVID-19 by taking the regular precautions such as staying home as much as possible and washing hands regularly. The guidelines also advise people to continue taking their medication in order to maintain glucose control and to stock up on an additional 30-day supply of medication and supplies for monitoring blood glucose levels at home. However, there are no specific guidelines targeted at individuals with multiple comorbidities, such as obesity and CVD. As per the guidance given by the International Diabetes Federation in the context of COVID-19 pandemic, people with diabetes are among those high risk categories that can have serious illness (just like the flu) if they get the virus and it is best not to rush to the hospital, to avoid transmitting the virus to others and to allow priority arrangements to be made by medical personnel, if needed, instead of having to wait in line (40). The International Society for Pediatric and Adolescent Diabetes (ISPAD) has updated its guidelines recently on 19 March 2020 amidst the recent COVID-19 pandemic (41).

NHS clinical guidelines for the management of diabetic patients in COVID-19 recommend expedition of treatment and discharge of inpatients, and the use of virtual clinics and teleconsultations in primary and secondary care settings (42). However, guidelines fail to address the need for extra measures to be taken for care of patients with poor access and literacy with regards to technology. Moreover, elderly patients and those with chronic disability living in nursing homes or aged care facilities are at heightened risk of infection. These patients often have a high prevalence of comorbid cardiovascular and diabetes risk factors which makes them vulnerable during a pandemic such as COVID-19. Increasing reports of acts of microaggression, xenophobia and discrimination are surfacing since the inception of this pandemic. This is particularly relevant to specific populations such as south-Asians, who have high rates of diabetes (43).

## Current Approaches to the Management of Cardiovascular Patients

Current approaches to the management of this population aim to continue care of patients during COVID-19, while minimizing the risk of transmission to both healthcare workers and patients. The current protocol at academic medical centers in China for Acute Myocardial Infarction involves compulsory screening for fever and respiratory symptoms, and any patients with STEMI that have suspected or confirmed infection are treated with emergency intravenous thrombolysis, in the absence of contraindications (44). For cardiologists in the operating theater, strict guidelines regarding hand hygiene and personal protective equipment (PPE) are followed, and the number of people in operating theater is minimized (44). The American College of Cardiology urges the implementation of telehealth in all cardiology clinics (45). Other societies including European Society of Cardiology, British Cardiovascular Society, Cardiac Society of Australian and New Zealand (CSANZ), High Blood Pressure Research Council of Australia (HBPRCA), Australian National Heart Foundation (NHF) and Australian and New Zealand Society of Cardiac and Thoracic Surgeons (ANZSCTS) also recommend use of telehealth services (17–19).

With regard to the use of ACEi and ARBs, several societies have highlighted that due to the limited nature of the evidence on this matter, it is advisable that ongoing management with such medication may continue in patients with diabetes and hypertension, unless otherwise clinically contra-indicated as per the case profile (46–48). Ongoing studies will bring clarity on the use of ACEi and ARBs in COVID-19 patients with diabetes and hypertension. A recent study reported a higher prevalence of CVD and more than 7% of patients suffer myocardial injury from the infection (22% of the critically ill) (49). Though ACE2 serves as the main gateway for infection, the role of ACEi or ARBs requires further investigation. Myocardial injury is present in more than a quarter of critical cases and presents in two patterns: acute myocardial injury and dysfunction on presentation; and myocardial injury that develops as the severity of illness intensifies (49–52). The continuation of clinically indicated ACEi and ARB medications is recommended based on the available evidence at this time though there are a number of promising treatments under investigation, but none with proven clinical efficacy to date. COVID-19 is proved to pose a challenge for heart transplantation, impacting donor selection, immunosuppression, and post-transplant management (52).

## Risk of Life-Threatening Cardiac Arrhythmic Events

Growing evidence suggests that COVID-19 is burdened by a higher risk of life-threatening cardiac arrhythmic events, especially in group of patients with diabetes and/or obesity, with important implications for survival (53). These life-threatening arrhythmias are also related to inflammation that can increase the duration of ventricular repolarization (QTc interval) (54). A particular attention to inflammation and arrhythmias is important in these patients that are frequently affected by QTc prolongation (55, 56). Therefore, key electrocardiogram (ECG) parameters such as QTc interval should be monitored in this subgroup of patients. Surveillance of QTc could potentially reduce the number of drug-induced ventricular arrhythmias and sudden cardiac deaths (57). This is particularly relevant as “off-label” agents such as hydroxychloroquine, azithromycin and lopinavir/ritonavir are being increasingly used in post-exposure prophylaxis or treatment of COVID-19 patients (57, 58). These drugs are proven to increase the risk of QTc interval prolongation, ventricular tachycardia (*torsades de pointes*) and sudden cardiac death (58).

## Recommendations and Discussions

**Figure 1:**
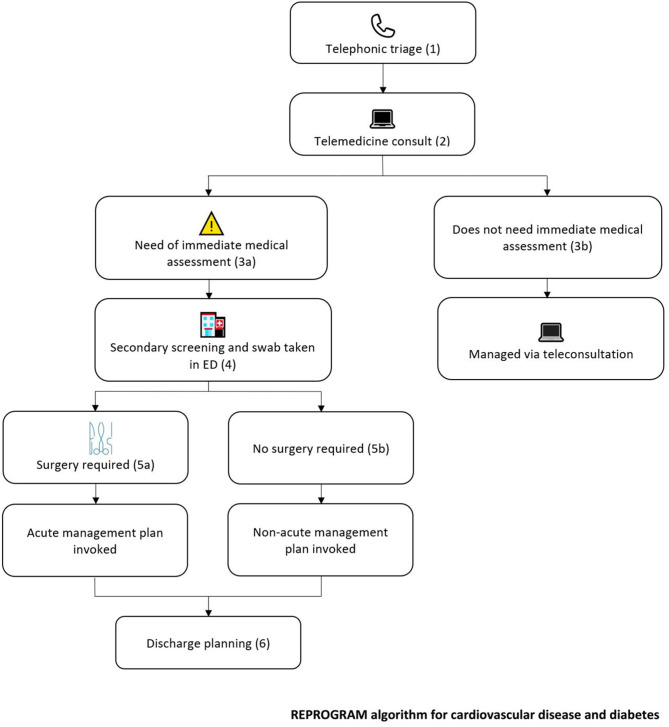
Proposed cardiovascular disease and diabetes risk-adjusted, stage-wise, tele and in-hospital triage and management protocol. (1) All patients seeking outpatient or in-hospital appointment, except the emergency cases, must dial into the hospital for a triage over telephone for risk assessment of COVID-19 cases prior to consultation. The triage will be carried out by the relevant department officer, and will comprise a brief screening for signs, symptoms, and risk factors of COVID-19. Questions should address recent travel history, fever, cough, sore throat, shortness of breath, fatigue, aches and pains, headaches, runny or stuffy nose, diarrhea, sneezing, and loss of smell. Patients should be screened for their body mass index (BMI) and those with BMI ≥ 30 should be closely monitored and strongly advised to self-isolate and follow public health guidelines. Patients with BMI ≥ 30 are at significant risk of mortality after COVID-19 infection. (2) All patients, despite risk factors and symptoms, should be asked to attend a compulsory teleconsultation in order to minimize harm to both the patient and consultant. During the consultation, further assessment of COVID-19 symptoms can be made, and potential impact on underlying diabetes/cardiovascular disease should be assessed. (3a) Should there be a self-reported acute emergency by the patient, or a need for immediate medical attention as per the clinical judgement of the physician, the patient should be asked to present at the emergency department; (b) If immediate medical assessment is not required, management should be carried out via teleconsultation. (4) In the emergency department, the relevant steward must carry out secondary screening for COVID-19 symptoms. After screening, patients should undergo diagnostic testing for COVID-19. Drive-through testing facilities should be deployed for all patients, to minimize exposure to health systems, health workers and the community. Further imaging should also be carried out on patients, with extra precautions being taken to ensure proper cleaning of equipment when imaging COVID-19 positive patients. (5a) For patients who require surgery, the acute management plan should be invoked. If the patient is COVID-19 positive, measures must be taken to protect healthcare workers involved. Minimal number of staff should be involved at the direct interface and risk-minimization should be ensured for any peri-surgical procedures that might involve aerosol production. For COVID-19 negative patients, the routine management plan should be followed; (b) If no surgery is required, the non-acute management plan should be invoked. (6) A plan should be made to ensure proper quarantine of patients after discharge. This could include home isolation and telemonitoring. Patients should be advised to follow hand hygiene, wear masks and practice social distancing.

Patients with diabetes with multiple comorbidities, such as obesity and CVD, should take extra precaution for the prevention of possible infection risk due to COVID-19. They are recommended to be in virtual contact with their primary health carers, and to maintain glycaemic control with diligence. To ensure the maintenance of adequate glucose control in such exceptional circumstances, it is recommended that primary health care physicians take additional interest/responsibility to reach out to patients who have not presented for regular check-ups. The main recommendations for pediatric and adolescents with diabetes and CVD are summarized (Table 1). Due to the alarming decline in patients presenting with emergent conditions to hospitals and outpatient clinics, without any indications of a fall in prevalence of these conditions, we would request public health professions to take extra measures in reaching out to patients regarding the safety of coming to hospitals and the medical need to do so and benefit of getting timely acute reperfusion therapy in eligible patients. Given the aggravated risks, we propose a novel triage and management protocol that takes into account risks with CVD and diabetes (Figure 1).

**Table 1:** Summary of recommendations regarding COVID-19 in patients with diabetes and/or cardiovascular disease.

**S. No**	**Stage of COVID-19 infection**	**Interventions/indications**
1	Prevention of infection and containing pandemic	1. Wash your hands frequently with soap and water for 20 s or clean with alcohol-based hand rub 2. Maintain social distancing (2 meters or 6 feet) 3. Cough or sneeze into tissue or elbow 4. Avoid touching your face 5. Sanitize surfaces frequently
2	Symptomatic stage	1. If the patient is feeling unwell, he/she should stay at home 2. If the patient has fever, cough and/or difficulty breathing, seek medical attention and call in advance 3. Follow the directions of your local health authority
3	Controlling diabetes during illness	General sick day diabetes management principles (modified from ISPAD guidelines): 1. More frequent blood glucose and ketone (blood or urine) monitoring 2. Aim for a blood glucose level between 4 and 10 mmol/L (70–180 mg/dL) and blood ketones below 0.6 mmol/L when the child is ill 3. NEVER STOP INSULIN: If there is FEVER, insulin needs are usually higher 4. Monitor and maintain hydration with adequate salt and water balance 5. Treat underlying illness and symptoms (fever)
4.	URGENT specialist advice/referral to emergency	1. Fever or vomiting persists and/or weight loss continues, suggesting worsening dehydration and potential circulatory compromise 2. Fruity breath odor (acetone) persists or worsens / blood ketones remain elevated >1.5 mmol/L or urine ketones remain large despite extra insulin and hydration 3. The patient is becoming exhausted, confused, hyperventilating (Kussmaul breathing), or has severe abdominal pain 4. Identify COVID-19 patients who are at high-risk of venous thromboembolism (VTE), including those with prolonged immobility, overlapping cardiovascular disease (CVD) risk factors (adiposity, age and smoking) or with high estrogen levels (including those on exogenous hormone therapy). Consider initiating appropriate prophylaxis. If at higher risk of bleeding due to anticoagulation, adjust anticoagulation dose and duration as well as use of mechanical compression 5. Patients with body mass index (BMI) of 30 kg/m^2^ or higher should be considered at high risk given the association of these patients with significantly higher mortality after COVID-19 infection. These patients need close monitoring over teleconsultation ^*^ 6. Patients who are at increased risk of QTc interval prolongation, life-threatening cardiac arrhythmic events and/or sudden cardiac death (e.g., COVID-19 positive patients with: (a) history of diabetes and/or CVD, and/or (b) those on post-exposure prophylaxis or treatment of COVID-19 using “off-label” drugs such as hydroxychloroquine, azithromycin and lopinavir/ritonavir)

*Source:Prepared and adapted by the authors from the ISPAD guidelines*.

*ISPAD: International Society for Pediatric and Adolescent Diabetes*.

* *Based on the analysis of Intensive Care National Audit & Research Centre (ICNARC) United Kingdom data set (analyzed on April 4, 2020)*.

^↑^*Recommendations of the CVD and diabetes subcommittee of the COVID-19 Pandemic Health System **RE**silience **PROGRAM (REPROGRAM)** consortium*.

Virtual delivery of group exercise classes could be organized for patients with diabetes, CVD or obesity, who are currently restricted by social isolation. Cross-department and peer-to-peer inter-specialty professional collaboration and communication are recommended to adapt existing pandemic preparedness and response strategies to manage patients with neuro cardiovascular emergencies. Special protection must be observed during interventions that produce aerosol (cardiopulmonary resuscitation). This may lower the risk of infection to healthcare workers and patients. Cardiovascular experts may brace themselves for deployments in different settings, for limited, extended or repurposed causes. The mobility of staff between COVID-19 treatment units and other patient facing consultation should be limited to avoid opportunities for nosocomial transmission. COVID-19 and patients with CVD, diabetes or obesity impact each other in compounding and negative dimensions. These patients are at increased risk of COVID-19 related hospitalization, morbidity and death; and those with COVID-19 also show propensity to increasing and emergent acute cardiovascular events. It is important to identify COVID-19 patients who are at high risk of VTE so that appropriate prophylaxis treatment could be initiated (22, 59). Anticoagulation should be considered for VTE prophylaxis. Given the high risk of bleeding in COVID-19 patients with high VTE risk, considerations should include adjustments in anticoagulant dose and duration as well as use of mechanical compressions (22).

Obesity is associated with severe COVID-19 (7–12, 22, 60). Moreover, obesity or higher BMI is known to be associated with a higher risk of CVD, diabetes and hypertension—which are independent predictors of poor outcomes in COVID-19 (50, 60). The analyses of ICNARC data suggest that BMI≥30 kg/m^2^ should be used as a prognostic indicator of mortality in critical care settings due to COVID-19 (22). Another recent study found a significant association of the prevalence of obesity (defined by BMI≥30 kg/m^2^) with severe COVID-19 (7). We recommend that clinicians should consider BMI≥30 while estimating risks and stratifying patients for early and ongoing intervention. Center for Disease Control and Prevention (CDC) in the United States also list obesity, although with a relatively higher BMI cut-off of ≥40 kg/m^2^, as an independent risk factor, of severe illness in COVID-19 (61). There are concerns this BMI cut-off (≥40 kg/m^2^) might mislead or compromise the safety of obese people at lower BMIs (60). The current consortium recommends BMI cut-off of 30 kg/m^2^ in identifying patients with adverse COVID-19 prognosis. Surveillance of ECG parameters is recommended to potentially reduce the risk of life-threatening arrhythmic events and sudden cardiac death in COVID-19 positive patients especially those with history of diabetes and/or obesity and/or those on post-exposure prophylactic treatment (53–58).

In addition to clinical management, public health interventions must be adhered to such as masks (preferably N95), washing hands, social distancing. A New England Journal of Medicine study showed efficacy of face masks in preventing further transmission of Coronavirus from symptomatic individuals (62). It is evident from the guidance currently issued between World Health Organization (WHO), the CDC in United States, the Canadian Standards Association and Canadian Federal guidance (Canada), and the UK that differences exist in advice for healthcare workers to use respirators as opposed to surgical face masks (63–65). The UK initially advocated, “COVID-19 is classified as an airborne high consequence infectious disease in the UK”, and instructed “ensure that staff who are assessing or caring for suspected COVID-19 cases are familiar with an FFP3 respirator conforming to EN149 [a protection level higher than N95], and that fit testing has been undertaken before using this equipment” (65). The current UK position aligns with WHO guidance although recommending risk assessment by the individual healthcare worker within the guidance framework. The situation for Low- and Middle-Income Countries is made more difficult by a lack of resources and the uncertain availability of respiratory and PPE often intended as single use only.

Recently the CDC recommended wearing cloth face coverings in public settings where other social distancing measures are difficult to maintain (e.g., grocery stores and pharmacies), especially in areas of significant community-based transmission (66). Coronavirus like influenza and rhinovirus can possibly spread through short range aerosol transmission in exhaled breath. Therefore, this study reinforces the need for individual and public health strategies and the adoption of using face masks as a preventive intervention. The American Academy of Ophthalmology (AAO) recommends contact lens wearers to switch to wearing glasses for a while to limit the risk of COVID-19 infection (67).

Pandemics like COVID-19, SARS and Spanish flu invoke irrational and heightened fear which could be linked to incidents of xenophobia and discrimination (68). A public health crisis of this scale can quickly mutate into a social and political crisis. Therefore, it is warranted that the political and health systems leadership must continue transparent, open, and respectful communication with all communities, with special consideration for communities from marginalized and vulnerable backgrounds, as they tend to have a disproportionately poor cardiovascular and metabolic profile (68). Also, this subgroup of patients often have relatively poor access to health services and compromised provision of medical supplies, which is exacerbated in a public health crisis situation, more so for a sustained period as is the case with pandemics such as COVID-19 with an estimated mortality of 3.4% however the recent evidence suggests the rates are still evolving (69, 70).

Patients with comorbid CVD, diabetes and obesity are potentially vulnerable in a pandemic (68–70). It must be considered that a significant number of healthcare workers will have these same and other vulnerabilities due to pre-existing health conditions, therefore institutional policies should provide for redeployment away from COVID-19 patient direct contact or furlough. Some jurisdictions have developed national policy or workplace sector guidance in others there is likely a duty of care in law. Healthcare providers, health systems and political leadership must account for the heterogeneity, compounded infection and fatality risks, long-term complications and special considerations for ongoing management as well as the socio-economic factors that may interfere with the health and well-being of patients with CVD, diabetes and/or obesity. Technological innovation such as telemedicine along with public health strategies may mitigate some of these risks.

## Data Availability Statement

The original contributions presented in the study are included in the article/supplementary material, further inquiries can be directed to the corresponding author/s.

## Author's Note

^†^The COVID19 pandemic is causing an unprecedented public health crisis impacting healthcare systems, healthcare workers and communities. The COVID-19 Pandemic Health System **RE**silience **PROGRAM (REPROGRAM)** consortium is a think-tank of leading international healthcare physicians, researchers and policymakers formed to champion the safety of healthcare workers, policy development and advocacy for global pandemic preparedness and action.

## Author Contributions

SBh devised the project, the main conceptual ideas and proof outline. SBh and AR wrote the first draft of the manuscript. SBh encouraged AR to investigate and supervised the findings of this work. All authors discussed the results and recommendations, and contributed to the final manuscript.

## Conflict of Interest

The authors declare that the research was conducted in the absence of any commercial or financial relationships that could be construed as a potential conflict of interest.

## References

[1] John Hopkins University COVID-19 Global Cases by the Center for Systems Science and Engineering (CSSE). Johns Hopkins University (2020). Available online at: https://coronavirus.jhu.edu/map.html (accessed April 6, 2020).

[2] Yang X Yu Y Xu J Shu H Xia Ja Liu H. Clinical course and outcomes of critically ill patients with SARS-CoV-2 pneumonia in Wuhan, China: a single-centered, retrospective, observational study. Lancet Respir Med. (2020) 8:475–81. 10.1016/S2213-2600(20)30079-5 32105632 PMC7102538

[3] Guan W-j Ni Z-y Hu Y Liang W-h Ou C-q He J-x. Clinical Characteristics of Coronavirus Disease 2019 in China. N Engl J Med. (2020) 382:1708–20. 10.1056/NEJMoa2002032 32109013 PMC7092819

[4] Zhang JJ Dong X Cao YY Yuan YD Yang YB Yan YQ Clinical characteristics of 140 patients infected with SARS-CoV-2 in Wuhan, China. Allergy. (2020). 10.1111/all.14238. [Epub ahead of print]. 32077115

[5] Wu Z McGoogan JM. Characteristics of and Important Lessons from the Coronavirus Disease 2019 (COVID-19) Outbreak in China: summary of a report of 72 314 cases from the Chinese Center for Disease Control and Prevention. JAMA. (2020) 323:1239–42. 10.1001/jama.2020.2648 32091533

[6] Yang J Zheng Y Gou X Pu K Chen Z Guo Q Prevalence of comorbidities and its effects in patients infected with SARS-CoV-2: a systematic review and meta-analysis. Int J Infect Dis. (2020) 94:91–5. 10.1016/j.ijid.2020.03.017 32173574 PMC7194638

[7] Caussy C Pattou F Wallet F Simon C Chalopin S Telliam C Prevalence of obesity among adult inpatients with COVID-19 in France. Lancet Diab Endocrinol. (2020). 10.1016/S2213-8587(20)30160-1. [Epub ahead of print]. PMC7234780 32437642

[8] Simonnet A Chetboun M Poissy J Raverdy V Noulette J Duhamel A High prevalence of obesity in severe acute respiratory syndrome coronavirus-2 (SARS-CoV-2) requiring invasive mechanical ventilation. Obesity. (2020). 10.1002/oby.22831. [Epub ahead of print]. PMC7262326 32271993

[9] Bhatraju PK Ghassemieh BJ Nichols M Kim R Jerome KR Nalla AK Covid-19 in critically ill patients in the seattle region - case series. N Engl J Med. (2020) 382:2012–22. 10.1056/NEJMoa2004500 32227758 PMC7143164

[10] Mahase E Covid-19: most patients require mechanical ventilation in first 24 hours of critical care. BMJ. (2020) 368:m1201 10.1136/bmj.m1201 32209544

[11] Chen Q Zheng Z Zhang C Zhang X Wu H Wang J. Clinical characteristics of 145 patients with corona virus disease 2019 (COVID-19) in Taizhou, Zhejiang, China. Infection. (2020). 10.1007/s15010-020-01432-5. [Epub ahead of print]. 32342479 PMC7186187

[12] Lighter J Phillips M Hochman S Sterling S Johnson D Francois F. Obesity in patients younger than 60 years is a risk factor for COVID-19 hospital admission. Clin Infect Dis. (2020). 10.1093/cid/ciaa415. [Epub ahead of print]. 32271368 PMC7184372

[13] Barclay L Nyarko E Are Diabetes, CVD Associated with Worse COVID-19 Prognosis? Medscape (2020). Available online at: https://www.medscape.org/viewarticle/926097 (accessed April 5, 2020).

[14] Fang L Karakiulakis G Roth M. Are patients with hypertension and diabetes mellitus at increased risk for COVID-19 infection? Lancet Respir Med. (2020) 8:e21. 10.1016/s2213-2600(20)30116-8 32171062 PMC7118626

[15] Hruby A Hu FB. The epidemiology of obesity: a big picture. Pharmacoeconomics. (2015) 33:673–89. 10.1007/s40273-014-0243-x 25471927 PMC4859313

[16] Leon BM Maddox TM. Diabetes and cardiovascular disease: Epidemiology, biological mechanisms, treatment recommendations and future research. World J Diabetes. (2015) 6:1246–58. 10.4239/wjd.v6.i13.1246 26468341 PMC4600176

[17] European Society of Cardiology (ESC) Available online at: https://www.escardio.org/Education/COVID-19-and-Cardiology (accessed May 28, 2020).

[18] British Cardiovascular Society (BCS) Available online at: https://www.britishcardiovascularsociety.org/resources/covid-19-clinicians-hub (accessed May 28, 2020).

[19] Zaman S MacIsaac AI Jennings GL Schlaich M Inglis SC Arnold R Cardiovascular disease and COVID-19: Australian/New Zealand consensus statement. Med J Aust [preprint]. (2020) 19. Available online at: https://www.mja.com.au/journal/2020/cardiovascular-disease-and-covid-19-australiannew-zealand-consensus-statement 10.5694/mja2.50714 32734645

[20] Gregson J Kaptoge S Bolton T Pennells L Willeit P Burgess S. Cardiovascular risk factors associated with venous thromboembolism. JAMA Cardiol. (2019) 4:163–73. 10.1001/jamacardio.2018.4537 30649175 PMC6386140

[21] Wang T Chen R Liu C Liang W Guan W Tang R. Attention should be paid to venous thromboembolism prophylaxis in the management of COVID-19. Lancet Haematol. (2020) 7:e362–3. 10.1016/s2352-3026(20)30109-5 32278361 PMC7158946

[22] Intensive Care National Audit and Research Centre Report on 2249 patients critically ill with COVID-19. Intensive Care National Audit and Research Centre (2020). Available online at: https://www.icnarc.org/About/Latest-News/2020/04/04/Report-On-2249-Patients-Critically-Ill-With-Covid-19 (accessed April 9, 2020).

[23] Ishiyama Y Gallagher PE Averill DB Tallant EA Brosnihan KB Ferrario CM. Upregulation of angiotensin-converting enzyme 2 after myocardial infarction by blockade of angiotensin II receptors. Hypertension. (2004) 43:970–6. 10.1161/01.HYP.0000124667.34652.1a 15007027

[24] Ferrario CM Jessup J Chappell MC Averill DB Brosnihan KB Tallant EA. Effect of angiotensin-converting enzyme inhibition and angiotensin II receptor blockers on cardiac angiotensin-converting enzyme 2. Circulation. (2005) 111:2605–10. 10.1161/circulationaha.104.510461 15897343

[25] Ocaranza MP Palomera C Román M Bargetto J Lavandero S Jalil JE. Effect of hypertension on angiotensin-(1-7) levels in rats with different angiotensin-I converting enzyme polymorphism. Life Sci. (2006) 78:1535–42. 10.1016/j.lfs.2005.07.026 16229862

[26] Klimas J Olvedy M Ochodnicka-Mackovicova K Kruzliak P Cacanyiova S Kristek F. Perinatally administered losartan augments renal ACE2 expression but not cardiac or renal Mas receptor in spontaneously hypertensive rats. J Cell Mol Med. (2015) 19:1965–74. 10.1111/jcmm.12573 25766467 PMC4549047

[27] Walters TE Kalman JM Patel SK Mearns M Velkoska E Burrell LM. Angiotensin converting enzyme 2 activity and human atrial fibrillation: increased plasma angiotensin converting enzyme 2 activity is associated with atrial fibrillation and more advanced left atrial structural remodelling. Europace. (2017) 19:1280–7. 10.1093/europace/euw246 27738071

[28] Burchill LJ Velkoska E Dean RG Griggs K Patel SK Burrell LM. Combination renin-angiotensin system blockade and angiotensin-converting enzyme 2 in experimental myocardial infarction: implications for future therapeutic directions. Clin Sci. (2012) 123:649–58. 10.1042/cs20120162 22715807

[29] Burrell LM Risvanis J Kubota E Dean RG MacDonald PS Lu S. Myocardial infarction increases ACE2 expression in rat and humans. Eur Heart J. (2005) 26:369–75. 10.1093/eurheartj/ehi114 15671045

[30] Witko-Sarsat V Rieu P Descamps-Latscha B Lesavre P Halbwachs-Mecarelli L. Neutrophils: molecules, functions and pathophysiological aspects. Lab Invest. (2000) 80:617–53. 10.1038/labinvest.3780067 10830774

[31] Gavin C Meinke S Heldring N Heck KA Achour A Iacobaeus E. The complement system is essential for the phagocytosis of mesenchymal stromal cells by monocytes. Front Immunol. (2019) 10:2249. 10.3389/fimmu.2019.02249 31616424 PMC6763726

[32] Medscape Glucose Control Key With COVID-19 in Diabetes, Say Experts. Medscape (2020). Available online at: https://www.medscape.com/viewarticle/927044 (accessed April 5, 2020).

[33] Camporotondo R Totaro R Costantino I Gnecchi M Oltrona L Patients: Scared and Alone. Pavia (2020). Available online at: https://www.escardio.org/Education/COVID-19-and-Cardiology/patients-scared-and-alone-pavia-italy (accessed April 5, 2020).

[34] Bhaskar S Sharma D Walker AH McDonald M Huasen B Haridas A Acute neurological care in the COVID-19 Era: the Pandemic Health System REsilience PROGRAM (REPROGRAM) Consortium Pathway. Front Neurol. (2020) 11:579 10.3389/fneur.2020.00579 PMC7273748 32574252

[35] American College of Surgeons COVID-19 Guidelines for Triage of Vascular Surgery Patients. American College of Surgeons (2020). Available online at: https://www.facs.org/covid-19/clinical-guidance/elective-case/vascular-surgery (accessed April 5, 2020).

[36] Colberg SR Sigal RJ Yardley JE Riddell MC Dunstan DW Dempsey PC. Physical activity/exercise and diabetes: a position statement of the American Diabetes Association. Diabetes Care. (2016) 39:2065–79. 10.2337/dc16-1728 27926890 PMC6908414

[37] The Lancet. COVID-19: protecting health-care workers. Lancet. (2020) 395:922. 10.1016/s0140-6736(20)30644-9 32199474 PMC7138074

[38] American Association of Clinical Endocrinologists AACE Position Statement: Coronavirus (COVID-19) and People with Diabetes. American Association of Clinical Endocrinologists (2020). Available online at: https://www.aace.com/recent-news-and-updates/aace-position-statement-coronavirus-covid-19-and-people-diabetes-updated (accessed April 5, 2020).

[39] European Society of Endocrinology A Statement from the European Society of Endocrinology COVID-19 and Endocrine Diseases. European Society of Endocrinology (2020). Available online at: https://www.ese-hormones.org/about-us/our-communities/clinicians/covid-19-and-endocrine-disease-clinical-information-and-comment-from-ese/ (accessed April 5, 2020).

[40] International Diabetes Federation (IDF) COVID-19 Outbreak: Guidance for People with Diabetes. International Diabetes Federation (2020). Available online at: https://www.idf.org/our-network/regions-members/europe/europe-news/196-information-on-corona-virus-disease-2019-covid-19-outbreak-and-guidance-for-people-with-diabetes.html (accessed April 7, 2020).

[41] International Society for Pediatric and Adolescent Diabetes (ISPAD) Coronavirus Infection (COVID-19) and Summary of Recommendations Regarding COVID-19 in Children with Diabetes. International Society for Pediatric and Adolescent Diabetes (ISPAD) (2020). Available online at: https://www.ispad.org/page/CoronavirusinfectionCOVID-19 (accessed April 7, 2020).

[42] National Health Service (NHS) UK Clinical Guide for the Management of People with Diabetes During the Coronavirus Pandemic. National Health Service (NHS) UK (2020). Available online at: https://www.england.nhs.uk/coronavirus/wp-content/uploads/sites/52/2020/03/speciality-guide-diabetes-19-march-v2-updated.pdf (accessed April 5, 2020).

[43] Devakumar D Shannon G Bhopal SS Abubakar I. Racism and discrimination in COVID-19 responses. Lancet. (2020) 395:1194. 10.1016/S0140-6736(20)30792-3 32246915 PMC7146645

[44] Jing Z-C Zhu H-D Yan X-W Chai W-Z Zhang S. Recommendations from the Peking Union Medical College Hospital for the management of acute myocardial infarction during the COVID-19 outbreak. Eur Heart J. (2020) 41:1791–4. 10.1093/eurheartj/ehaa258 32232396 PMC7184505

[45] American College of Cardiology Telehealth: Rapid Implementation for Your Cardiology Clinic. American College of Cardiology (2020). Available online at: https://www.acc.org/latest-in-cardiology/articles/2020/03/01/08/42/feature-telehealth-rapid-implementation-for-your-cardiology-clinic-coronavirus-disease-2019-covid-19 (accessed April 5, 2020).

[46] European Society of Cardiology Position Statement of the ESC Council on Hypertension on ACE-Inhibitors and Angiotensin Receptor Blockers. European Society of Cardiology (2020). Available online at: https://www.escardio.org/Councils/Council-on-Hypertension-(CHT)/News/position-statement-of-the-esc-council-on-hypertension-on-ace-inhibitors-and-ang (accessed April 5, 2020).

[47] American Heart Association Patients Taking ACE-i and ARBs Who Contract COVID-19 Should Continue Treatment, Unless Otherwise Advised by Their Physician. American Heart Association (2020). Available online at: https://newsroom.heart.org/news/patients-taking-ace-i-and-arbs-who-contract-covid-19-should-continue-treatment-unless-otherwise-advised-by-their-physician (accessed April 5, 2020).

[48] European Society of Hypertension European Society of Hypertension Update on COVID-19. European Society of Hypertension (2020). Available online at: https://www.eshonline.org/spotlights/esh-stabtement-on-covid-19/ (accessed April 5, 2020).

[49] Wang D Hu B Hu C Zhu F Liu X Zhang J. Clinical characteristics of 138 hospitalized patients with 2019 novel coronavirus–infected pneumonia in Wuhan, China. JAMA. (2020) 323:1061–9. 10.1001/jama.2020.1585 32031570 PMC7042881

[50] Zhou F Yu T Du R Fan G Liu Y Liu Z. Clinical course and risk factors for mortality of adult inpatients with COVID-19 in Wuhan, China: a retrospective cohort study. Lancet. (2020) 395:1054–62. 10.1016/s0140-6736(20)30566-3 32171076 PMC7270627

[51] Zheng Y-Y Ma Y-T Zhang J-Y Xie X. COVID-19 and the cardiovascular system. Nat Rev Cardiol. (2020) 17:259–60. 10.1038/s41569-020-0360-5 32139904 PMC7095524

[52] Clerkin KJ Fried JA Raikhelkar J Sayer G Griffin JM Masoumi A. COVID-19 and cardiovascular disease. Circulation. (2020) 141:1648–55. 10.1161/CIRCULATIONAHA.120.046941 32200663

[53] Baldi E Sechi GM Mare C Canevari F Brancaglione A Primi R. Out-of-hospital cardiac arrest during the covid-19 outbreak in Italy. N Engl J Med. (2020). 10.1056/NEJMc2010418. [Epub ahead of print]. 32348640 PMC7204428

[54] Lazzerini PE Boutjdir M Capecchi PL. COVID-19, arrhythmic risk and inflammation: mind the gap! Circulation. (2020). 10.1161/CIRCULATIONAHA.120.047293. [Epub ahead of print]. 32286863

[55] Omran J Bostick BP Chan AK Alpert MA. Obesity and ventricular repolarization: a comprehensive review. Prog Cardiovasc Dis. (2018) 61:124–35. 10.1016/j.pcad.2018.04.004 29698642

[56] Kobayashi S Nagao M Asai A Fukuda I Oikawa S Sugihara H. Severity and multiplicity of microvascular complications are associated with QT interval prolongation in patients with type 2 diabetes. J Diabetes Investig. (2018) 9:946–51. 10.1111/jdi.12772 29095573 PMC6031516

[57] Giudicessi JR Noseworthy PA Friedman PA Ackerman MJ. Urgent guidance for navigating and circumventing the QTc-prolonging and torsadogenic potential of possible pharmacotherapies for Coronavirus Disease 19 (COVID-19). Mayo Clin Proc. (2020). 10.1016/j.mayocp.2020.03.024. [Epub ahead of print]. 32359771 PMC7141471

[58] Chorin E Dai M Shulman E Wadhwani L Bar-Cohen R Barbhaiya C The QT interval in patients with COVID-19 treated with hydroxychloroquine and azithromycin. Nat Med. (2020). 10.1038/s41591-020-0888-2. [Epub ahead of print]. 32488217

[59] Barbar S Noventa F Rossetto V Ferrari A Brandolin B Perlati M. A risk assessment model for the identification of hospitalized medical patients at risk for venous thromboembolism: the Padua Prediction Score. J Thromb Haemost. (2010) 8:2450–7. 10.1111/j.1538-7836.2010.04044.x 20738765

[60] Flint SW Tahrani AA. COVID-19 and obesity lack of clarity, guidance, and implications for care. Lancet Diab Endocrinol. (2020) 8:474–5. 10.1016/S2213-8587(20)30156-X 32359411 PMC7190298

[61] Government of UK Guidance: Staying Alert and Safe (Social Distancing). Government of UK (2020). Available online at: https://www.gov.uk/government/publications/staying-alert-and-safe-social-distancing/staying-alert-and-safe-social-distancing#clinically-vulnerable-people (accessed May 28, 2020).

[62] Leung NHL Chu DKW Shiu EYC Chan K-H McDevitt JJ Hau BJP. Respiratory virus shedding in exhaled breath and efficacy of face masks. Nat Med. (2020) 26:676–80. 10.1038/s41591-020-0843-2 32371934 PMC8238571

[63] World Health Organization Infection Prevention and Control During Health Care when Novel Coronavirus (nCoV) Infection Is Suspected. World Health Organization (2020). Available online at: https://www.who.int/publications-detail/infection-prevention-and-control-during-health-care-when-novel-coronavirus-(ncov)-infection-is-suspected-20200125 (accessed April 6, 2020).

[64] Public Health Agency of Canada Infection Prevention and Control for Novel Coronavirus (2019-nCoV): Interim Guidance for Acute Healthcare Settings. Public Health Agency of Canada (2020). Available online at: https://www.canada.ca/en/public-health/services/diseases/2019-novel-coronavirus-infection/health-professionals/interim-guidance-acute-healthcare-settings.html#a4.10 (accessed April 6, 2020).

[65] Public Health England Guidance on Infection Prevention and Control for COVID-19. Public Health England (2020). Available online at: https://assets.publishing.service.gov.uk/government/uploads/system/uploads/attachment_data/file/866112/COVID-19_Donning_guidance_web_v1_14_February_2020.pdf (accessed April 6, 2020).

[66] The Center for Disease Control and Prevention (CDC) Use of Cloth Face Coverings to Help Slow the Spread of COVID-19. The Center for Disease Control and Prevention (CDC) (2020). Available online at: https://www.cdc.gov/coronavirus/2019-ncov/prevent-getting-sick/diy-cloth-face-coverings.html (accessed on April 6, 2020).

[67] American Academy of Ophthalmology (AAO) Coronavirus Eye Safety. American Academy of Ophthalmology (AAO) (2020). Available online at: https://www.aao.org/eye-health/tips-prevention/coronavirus-covid19-eye-infection-pinkeye (accessed April 7, 2020).

[68] Ahorsu DK Lin C-Y Imani V Saffari M Griffiths MD Pakpour AH. The fear of COVID-19 Scale: development and initial validation. Int J Ment Health Addict. (2020) 1–9. 10.1007/s11469-020-00270-8 32226353 PMC7100496

[69] García-Basteiro AL Chaccour C Guinovart C Llupià A Brew J Trilla A. Monitoring the COVID-19 epidemic in the context of widespread local transmission. Lancet Respir Med. (2020) 8:440–2. 10.1016/S2213-2600(20)30162-32247325 PMC7198847

[70] World Health Organization Director-General's Opening Remarks at the Media Briefing on COVID-19. World Health Organization (2020). Available online at: https://www.worldometers.info/coronavirus/coronavirus-death-rate/#ref-13 (accessed April 11, 2020).

